# Higher incidence of acute symptomatic seizures in probable antibody-negative pediatric autoimmune encephalitis than in major antibody-positive autoimmune encephalitis

**DOI:** 10.3389/fneur.2024.1418083

**Published:** 2024-07-19

**Authors:** Naoki Yamada, Takeshi Inoue, Ichiro Kuki, Naohiro Yamamoto, Masataka Fukuoka, Megumi Nukui, Hideo Okuno, Junichi Ishikawa, Kiyoko Amo, Masao Togawa, Hiroshi Sakuma, Shin Okazaki

**Affiliations:** ^1^Department of Pediatric Neurology and Logopedics, Osaka City General Hospital, Osaka, Japan; ^2^Department of Pediatric Emergency, Infectious Disease Internal Medicine, Osaka City General Hospital, Osaka, Japan; ^3^Department of Brain and Neuroscience, Tokyo Metropolitan Institute of Medical Science, Tokyo, Japan

**Keywords:** pediatric, autoimmune encephalitis, antibody-negative, seizure, epilepsy

## Abstract

**Purpose:**

To delineate the characteristics of probable antibody-negative pediatric autoimmune encephalitis (probable Ab-negative AE), we compared the clinical features of probable Ab-negative AE to those of major antibody-positive AE.

**Methods:**

We retrospectively reviewed the clinical features of 18 patients with probable Ab-negative AE, 13 with anti-N-methyl-D-aspartate receptor encephalitis (NMDARE), and 13 with myelin oligodendrocyte glycoprotein antibody-associated disease (MOGAD). Clinical characteristics, neuroimaging findings, treatments, and outcomes were analyzed.

**Results:**

The age of onset and length of hospital stay were significantly higher in the NMDARE group than in the other groups (*p* = 0.02 and *p* < 0.01). Regarding initial neurological symptoms, acute symptomatic seizures in the probable Ab-negative AE group (67%) were significantly more frequent than in the NMDARE (15%) and MOGAD (23%) groups (*p* < 0.01). Paraclinical evidence of neuroinflammation within 1 month of disease onset revealed that single-photon emission computed tomography (SPECT) detected abnormal alterations in 14/14 (100%), cerebrospinal fluid (CSF) analysis in 15/18 (83%), and magnetic resonance imaging (MRI) in 11/18 (61%) in patients with probable Ab-negative AE. In the probable Ab-negative AE group, seven patients (39%) developed autoimmune-associated epilepsy, whereas one patient (8%) had both NMDARE and MOGAD (not statistically significant, *p* = 0.07).

**Conclusion:**

Patients with probable Ab-negative AE exhibited acute symptomatic seizures as initial neurological symptoms significantly more frequently. They developed autoimmune-associated epilepsy more frequently than those with NMDARE and MOGAD, which was not statistically significant. SPECT within 1 month of disease onset might be a valuable surrogate marker of ongoing neuroinflammation and neuronal dysfunction, even in patients with negative MRI findings.

## 1 Introduction

Autoimmune encephalitis (AE) is a common disease worldwide. A series of neuronal autoantibodies in the central nervous system has been demonstrated to be involved in a subset of AE (1–7). However, neuronal autoantibody tests are not readily available in most institutions in Japan, and obtaining the results may take several weeks or more, potentially delaying appropriate therapeutic intervention. Notably, a practical syndrome-based diagnostic approach for AE in adults emphasizes that immunotherapy should be started based on the initial neurological assessment and conventional laboratory investigations without waiting for neuronal autoantibody results (3, 5, 8, 9).

Several antibodies have been detected in a subset of pediatric patients with AE (3, 10–13). Among these, the most common extracellular antibodies in pediatric patients target N-methyl-D-aspartate receptor (NMDAR) (1, 13–15) and myelin oligodendrocyte glycoprotein (MOG) (16–18). However, it is essential to note that not all pediatric patients with AE have known autoantibodies, and the diagnosis of antibody-negative AE in pediatric patients can be challenging because of their diverse clinical features, often leading to delays in therapeutic interventions (3).

To address these problems mentioned above, a subcommittee of the Autoimmune Encephalitis International Working Group proposed provisional classification criteria for possible AE, probable antibody-negative AE (probable Ab-negative AE), and definite antibody-positive AE in pediatric patients (10). These criteria emphasize that the diagnosis of pediatric AE is based on a combination of clinical features and supportive diagnostic investigations, which include but are not solely dependent on the results of neuronal autoantibody tests. Pediatric patients with a clinical phenotype of AE and paraclinical findings of neuroinflammation but are negative for neural antibodies may be classified as having probable Ab-negative AE. To our knowledge, few comparative studies have been conducted to investigate the clinical features and outcomes of Ab-negative and Ab-positive AE in pediatric patients (19–21).

This study aimed to elucidate the characteristics of probable Ab-negative AE in pediatric patients. We compared the clinical features, diagnostic investigations, immunotherapies, and outcomes between probable Ab-negative AE and NMDAR antibody encephalitis (NMDARE) or MOG-associated disease (MOGAD).

## 2 Materials and methods

### 2.1 Patients

Eighteen consecutive pediatric patients (age < 18 years) with a diagnosis of probable Ab-negative AE who were referred to Osaka City General Hospital between 1 January 2015 and 31 March 2023 were included in this study. To establish meaningful comparisons, we also included two control groups: 13 patients with NMDARE from 1 January 2002 to 31 March 2023 and 13 patients with MOGAD from 1 January 2015 to 31 March 2023.

### 2.2 Definitions of probable Ab-negative AE, NMDARE, and MOGAD

The diagnosis of AE was based on criteria from a recent report on provisional classification criteria proposed by a subcommittee of the Autoimmune Encephalitis International Working Group (10), with slight modifications.

The criteria for probable Ab-negative AE are presented as follows:

1. Evidence of acute or subacute onset of neurological and/or psychiatric symptoms over 3 months in a previously healthy child.
2. Clinical evidence of neurological dysfunctions, including two or more features from the following list: (i) acute symptomatic seizures (ASSs), (ii) altered mental status/level of consciousness, (iii) cognitive difficulties/acute developmental regression, (iv) psychiatric symptoms, (v) focal neurological deficits, and (vi) movement disorders (except tics). Cognitive difficulties and acute developmental regression, which Cellucci et al. evaluated separately, were considered together in this study.
3. Paraclinical evidence of neuroinflammation confirmed within 1 month of disease onset, including one or more of the following features: cerebrospinal fluid (CSF) inflammatory changes [leukocytosis >5/μL, proteins >42 mg/dL, and oligoclonal banding (OCB)], magnetic resonance imaging (MRI) features of encephalitis, and increased/decreased blood flow on single-photon emission computed tomography (SPECT).
4. Negative serum and CSF results for well-characterized autoantibodies associated with AE before immunotherapy. NMDAR and anti-MOG antibodies were examined in the serum using in-house cell-based assays. In the CSF, the following autoantibodies were assessed using a cell-based indirect immunofluorescence assay (Autoimmune Encephalitis Mosaic 1, Euroimmun, Lübeck, Germany) according to the manufacturer’s instructions (14): NMDAR, anti-leucine-rich glioma-inactivated protein 1 (LGI1), anti-contactin-associated protein 2 (Casper2), anti-a-amino-3-hydroxy-5-methyl-4-isoxazole propionic acid receptor 1 and 2 (AMPAR1/2), and anti-g-aminobutyric acid type B receptor (GABABR). Three patients presented with unique MRI lesions in the basal ganglia and periventricular white matter and underwent examination for anti-glial fibrillary acidic protein (GFAP) antibodies. Among them, one patient was diagnosed with autoimmune GFAP astrocytopathy and was consequently excluded from this study (12), while two with negative findings were included in this study.
5. Exclusion of the following disorders: (i) infectious encephalitis and viral/bacterial meningitis. To rule out infectious encephalitis and viral/bacterial meningitis, we conducted a comprehensive investigation at our institution, which actively participates in viral surveillance in Osaka, Japan. The serum and CSF were examined for Herpes simplex virus type 1 and 2, human herpesvirus 6 and 7, Epstein–Barr virus, cytomegalovirus, and varicella zoster virus. No viral isolation was detected in the CSF, nasal discharge, or stool, which are potential sources of infectious encephalitis and viral meningitis. Furthermore, bacterial cultures in the blood and CSF yielded negative results; (ii) relatively well-established neuroinflammatory disorders, such as febrile illness-related epilepsy syndrome (22–24) and Rasmussen encephalitis (25); and (iii) primary psychiatric/neurological disorders, including pre-existing epilepsy.

The provisional classification criteria proposed by Cellucci et al. (10) categorize patients into three groups: possible AE, probable Ab-negative AE, and definite antibody-positive AE. Possible AE cases meet the criteria for (1) (clinical course) and (2) (clinical symptoms) but have not undergone (3) (CSF, MRI, and SPECT). Probable Ab-negative AE cases meet the criteria for (1), (2), and (3). At our institution, all patients who met the criteria for possible AE [(1, 2)] underwent (3), which were classified as probable Ab-negative AE. Subsequently, serum and CSF antibody tests were performed, and those that were found to be positive for NMDAR and anti-MOG antibodies were defined as definite antibody-positive AE. Hence, we considered that comparing probable Ab-negative AE with definite antibody-positive AE was clinically meaningful.

In the diagnosis of NMDARE and MOGAD, positive NMDAR antibodies in the CSF and MOG antibodies in the serum were confirmed. However, overlapping antibodies involving both NMDAR and MOG were detected in two patients, and they were subsequently excluded from this study. Regarding MOGAD, only the initial episode was evaluated because the second and subsequent recurrences might have been influenced by residual MRI lesions and symptoms following the initial episode.

### 2.3 Investigations

All patients underwent a comprehensive evaluation of clinical symptoms, CSF analysis, MRI, SPECT, and electroencephalography (EEG) within 1 month of disease onset. In SPECT, using technetium-99 ethyl cysteinate dimer (^99m^Tc-ECD) and easy Z-score imaging system (eZIS) analyses, we performed a computer-assisted statistical analysis, following anatomical standardization, based on a comparison with a normal database. eZIS analyses were used to supplement the diagnosis based on MRI findings and assess abnormal blood perfusion (26). A regional Z-score > 2.0 or < −2.0 standard deviation was considered significant. Recorded electroclinical seizures, interictal epileptic discharge, and changes in background activity (focal and generalized slow waves) were assessed. EEG was evaluated and confirmed by at least two pediatric neurologists. Radiological findings were reviewed by two pediatric neurologists and one radiologist to ensure accuracy and reliability.

### 2.4 Immunotherapies

First-line immunotherapy included the separate or combined use of intravenous methylprednisolone [IVMP, 30 mg/kg/day (maximum 1,000 mg/day) for 3 days] and intravenous immunoglobulin (IVIG, 0.4 g/kg for 5 days). If these immunotherapies were ineffective or the symptoms progressed rapidly, plasma exchange (PLEX, 3 consecutive days, followed by 4 consecutive days every other day) was added. If the symptoms did not improve, second-line immunotherapy was initiated with rituximab (375 mg/m^2^ weekly for 4 weeks). For patients with prolonged symptoms, oral prednisone (starting dose, 1 mg/kg/day with variable tapering durations) followed by IVMP was considered a maintenance treatment.

### 2.5 Antiseizure medications

Patients with ASS on admission were treated with intravenous midazolam (MDL) (0.1–0.3 mg/kg/dose), and those with ASS in clusters were administered continuous intravenous MDL (0.1–0.3 mg/kg/h), with video-EEG monitoring employed whenever possible. In addition, infusions of levetiracetam (LEV) (20–40 mg/kg/day) and fosphenytoin (initial dose: 22.5 mg/kg; maintenance dose: 7.5 mg/kg/day) were also added as needed. In patients whose ASS control was not achieved using the above measures, barbiturate therapy (continuous intravenous thiopental (3–5 mg/kg/h) with targeted temperature management at 36–37°C) was administered 48 h after intubation. ASMs, primarily LEV or other ASMs, were administered for ongoing ASS management.

### 2.6 Outcome assessments

Disease severity and treatment outcomes were assessed using the modified Rankin Scale (mRS) scores at the admission, the worst point during the disease course, and the final follow-up. A favorable outcome was defined as an mRS score of 0–2 at the final follow-up (27). In addition, the frequency of ASS within the first 3 months from the onset of AE and the transition to autoimmune-associated epilepsy at the last follow-up was also assessed. Autoimmune-associated epilepsy was defined as the presence of persistent non-provoked seizures without obvious evidence of active inflammation for more than 3 months from the onset of AE (28).

### 2.7 Statistical analysis

Continuous data were reported as median and interquartile range. Categorical data were presented as numbers and percentages. For categorical data, Fisher’s exact test was used to compare the three groups, followed by Fisher’s exact test between the two groups. For continuous quantity and ordinal data, the Kruskal–Wallis test was used to compare the three groups, followed by the Mann–Whitney U-test between the two groups. Within-group comparisons of ordinal variables were performed using the Wilcoxon signed-rank tests. The multiplicity of comparisons among groups was accounted for using the Bonferroni correction. Statistical significance was set at a *p-*value of < 0.05. All statistical analyses were performed using SPSS software (version 22.0; IBM Japan, Tokyo, Japan).

### 2.8 Ethics approval

This study was approved by the Ethics Committee of Osaka City General Hospital (No. 1508052). Written informed consent for this study and its publication was obtained from the parents of patients.

## 3 Results

### 3.1 Patients and clinical characteristics

**Figure 1:**
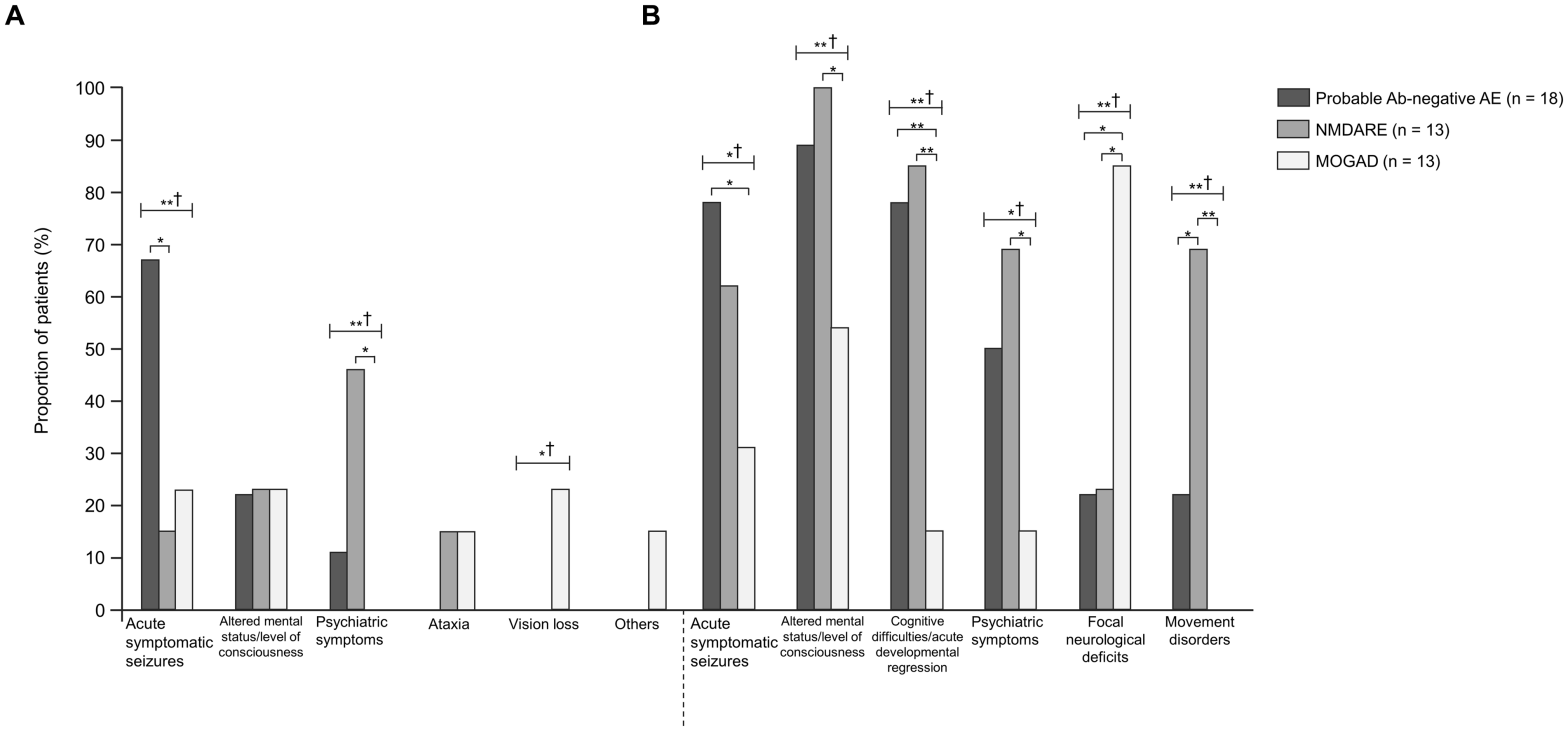
Comparisons between probable Ab-negative pediatric autoimmune encephalitis and major antibody-positive autoimmune encephalitis. **(A)** Initial neurological symptoms and **(B)** clinical evidence of neurologic dysfunction in the clinical course. † indicates Fisher’s exact test among the three groups. Fisher’s exact test between the two groups was then used. The multiplicity of comparisons among groups was accounted for using the Bonferroni correction. *p < 0.05 and **p < 0.01. MOGAD, myelin oligodendrocyte glycoprotein-associated disease; NMDARE, anti-N-methyl-D-aspartate receptor encephalitis; Probable Ab-negative AE, probable antibody-negative pediatric autoimmune encephalitis.

The age of onset and length of hospital stay were significantly higher in the NMDARE group than in the other groups (*p* = 0.02 and *p* < 0.01) (Table 1). Regarding initial neurological symptoms, the incidence of ASS (12/18, 67%) was significantly higher in the probable Ab-negative AE group than in the NMDARE (15%) and MOGAD (23%) groups (*p* < 0.01) and that of psychiatric symptoms (6/13, 46%) was significantly higher in the NMDARE group (*p* < 0.01) (Figure 1A). The most common symptoms were the altered mental status or level of consciousness in the probable Ab-negative AE (16/18, 89%) and NMDARE (13/13, 100%) groups and focal neurological deficits in the MOGAD group (11/13, 85%) (Figure 1B). Among the 14 patients (78%) with ASS in the probable Ab-negative AE group, 11 experienced ASS in clusters, 4 had status epilepticus, and 1 had both conditions. Three patients whose ASS occurred in clusters required barbiturate coma therapy for ASS control in the pediatric intensive care unit.

**Table 1:** Clinical characteristics of probable Ab-negative pediatric autoimmune encephalitis and major antibody-positive autoimmune encephalitis.

	Probable-Ab negative AE (Ab−, *n* = 18)	NMDARE (*N*, *n* = 13)	MOGAD (*M*, *n* = 13)	*p*-value	
Male (%)	12 (67%)	3 (23%)	6 (46%)	0.06	
Age at onset, median years (IQR)	8.1 (6.0, 11.0)	13.5 (11.3, 14.3)	6.4 (5.0, 11.5)	0.02*	N > Ab-
Hospital stay, median days (IQR)	30.5 (20.5, 42.5)	68 (43, 95)	28 (21, 37)	< 0.01**	N > Ab−, M
ICU admission (%)	3 (17%)	6 (46%)	0	< 0.01**	N > M
Follow-up duration, median years (IQR)	5.0 (3.5, 6.7)	14.4 (4.6, 15.1)	4.1 (2.8, 5.2)	0.04*	
Prodromal symptoms (%)	18 (100%)	10 (77%)	11 (85%)	0.09	
Fever (%)	17 (94%)	7 (54%)	10 (77%)	0.03*	Ab− > N
Headache (%)	6 (33%)	7 (54%)	6 (46%)	0.55	
Respiratory symptoms (%)	4 (22%)	1 (8%)	1 (8%)	0.47	
Initial prodromal symptoms to initial neurological symptoms, median days (IQR)	5.2 (2.2, 8.9)	3.0 (1.0, 5.4)	10 (5.0, 21.0)	0.07	
Initial neurological symptoms to first-line immunotherapy, median days (IQR)	1.1 (0.8, 6.7)	6.0 (3.0, 9.3)	6.0 (4.0, 8.0)	0.06	
Immunotherapy					
First-line immunotherapy					
IVMP (%)	18 (100%)	12 (92%)	13 (100%)	0.59	
IVIG (%)	14 (78%)	11 (85%)	10 (77%)	>0.99	
PLEX (%)	1 (6%)	3 (23%)	2 (15%)	0.40	
First- to second-line immunotherapy, days	86	29, 47, 79, 84			
Second-line immunotherapy (rituximab) (%)	1 (6%)	4 (31%)	0	0.05	
Oral prednisolone (%)	14 (78%)	10 (77%)	13 (100%)	0.17	
Intravenous ASMs					
Levetiracetam (%)	15 (83%)	6 (46%)	4 (31%)	<0.01**	Ab− > M
Midazolam (%)	7 (39%)	4 (31%)	0	0.04*	
Thiopental (%)	5 (28%)	2 (15%)	0	0.12	
Fosphenytoin (%)	3 (17%)	0	0	0.10	
Oral ASMs	16 (89%)	8 (62%)	5 (39%)	0.01*	Ab− > M
Good outcome (mRS 0–2)	17 (94%)	10 (77%)	13 (100%)	0.16	
Relapse (%)	0	1 (8%)	7 (54%)	<0.01**	M > Ab−, N
Epilepsy (%)	7 (39%)	1 (8%)	1 (8%)	0.07	
Drug-resistant epilepsy (%)	3 (17%)	0	0	0.10	

*p < 0.05 and **p < 0.01.

ASMs, antiseizure medications; ICU, intensive care unit; IQR, interquartile range; IVIG, intravenous immunoglobulin; IVMP, intravenous methylprednisolone; MOGAD (M), myelin oligodendrocyte glycoprotein-associated disease; mRS, modified Rankin Scale; NMDARE (N), anti-N-methyl-D-aspartate receptor encephalitis; PLEX, plasma exchange; Probable Ab-negative AE (Ab−), probable antibody-negative pediatric autoimmune encephalitis.

### 3.2 Paraclinical evidence of neuroinflammation

CSF and MRI findings were examined in all patients. ECD-SPECT was performed in 14 out of 18 (78%) patients with probable Ab-negative AE, 3 out of 13 (23%) patients with NMDARE, and 2 out of 13 (15%) patients with MOGAD within 1 month of disease onset (Table 2). Cell counts, protein levels, OCB positivity, and neopterin levels in the CSF were not significantly different among the three groups.

**Table 2:** Clinical findings of probable Ab-negative pediatric autoimmune encephalitis and major antibody-positive autoimmune encephalitis.

	Probable-Ab negative AE (Ab−, *n* = 18)	NMDARE (*N*, *n* = 13)	MOGAD (*M*, *n* = 13)	*p*-value	
**Paraclinical evidence of neuroinflammation within 1 month of disease onset**					
**Cerebrospinal fluid**					
Cell > 5/μL (%)	15/18 (83%)	12/13 (92%)	11/13 (85%)	0.87	
Cell counts, median (IQR)	17 (1–225)	24 (4–245)	41 (1–201)	0.76	
Protein (mg/dL), median (IQR)	28.5 (13–92)	25 (11–39)	38 (22–54)	0.11	
Oligoclonal banding positivity (%)	4/17 (24%)	7/11 (64%)	4/10 (40%)	0.12	
**MRI abnormalities**	11/18 (61%)	4/13 (31%)	13/13 (100%)	<0.01**	M > N
Medial temporal lobe (%)	9 (50%)	3 (23%)	1 (8%)	<0.01**	Ab− > M
Lateral temporal lobe (%)	1 (6%)	1 (8%)	7 (54%)	0.05*	
Frontal lobe (%)	2 (11%)	0	10 (77%)	<0.01**	M > Ab−, N
Parietal lobe (%)	0	0	6 (46%)	0.01*	M > Ab−
Occipital lobe (%)	0	0	6 (46%)	0.01*	M > Ab−
Basal ganglia (%)	2 (11%)	0	0	0.42	
Insula (%)	2 (11%)	0	0	0.42	
Brainstem (%)	1 (6%)	0	0	0.54	
Optic nerve	0	0	4 (31%)	0.13	
**ECD-SPECT abnormalities**	14/14 (100%)	3/3 (100%)	2/2 (100%)	–	
Medial temporal lobe (%)	11 (79%)	2 (67%)	0	0.11	
Lateral temporal lobe (%)	13 (93%)	2 (67%)	1 (50%)	0.15	
Frontal lobe (%)	13 (93%)	2 (67%)	1 (50%)	0.15	
Occipital lobe (%)	7 (50%)	2 (67%)	1 (50%)	>0.99	
Cerebellum	13 (93%)	2 (67%)	1 (50%)	0.15	
Brainstem (%)	9 (64%)	0	0	0.03*	
Basal ganglia (%)	8 (57%)	0	2 (100%)	0.07	
Insula (%)	8 (57%)	1 (33%)	0	0.47	
Caudate nucleus (%)	5 (36%)	1 (33%)	0	>0.99	
Thalamus (%)	5 (36%)	1 (33%)	0	>0.99	
**EEG abnormalities**	16/17 (94%)	9/10 (90%)	7/10 (70%)	0.23	
Recorded electroclinical seizure (%)	6 (35%)	3 (30%)	0	0.10	
Interictal epileptic discharges (%)	9 (53%)	2 (20%)	0	0.01*	Ab− > M
Focal slow waves (%)	15 (88%)	7 (70%)	6 (60%)	0.22	
Generalized slow waves (%)	16 (94%)	6 (60%)	3 (30%)	<0.01**	Ab− > M

*p < 0.05 and **p < 0.01.

ECD-SPECT, ethyl cysteinate dimer-single-photon emission computed tomography; MOGAD (M), myelin oligodendrocyte glycoprotein-associated disease; NMDARE (N), anti-N-methyl-D-aspartate receptor encephalitis; Probable Ab-negative AE (Ab−), probable antibody-negative pediatric autoimmune encephalitis.

**Figure 2:**
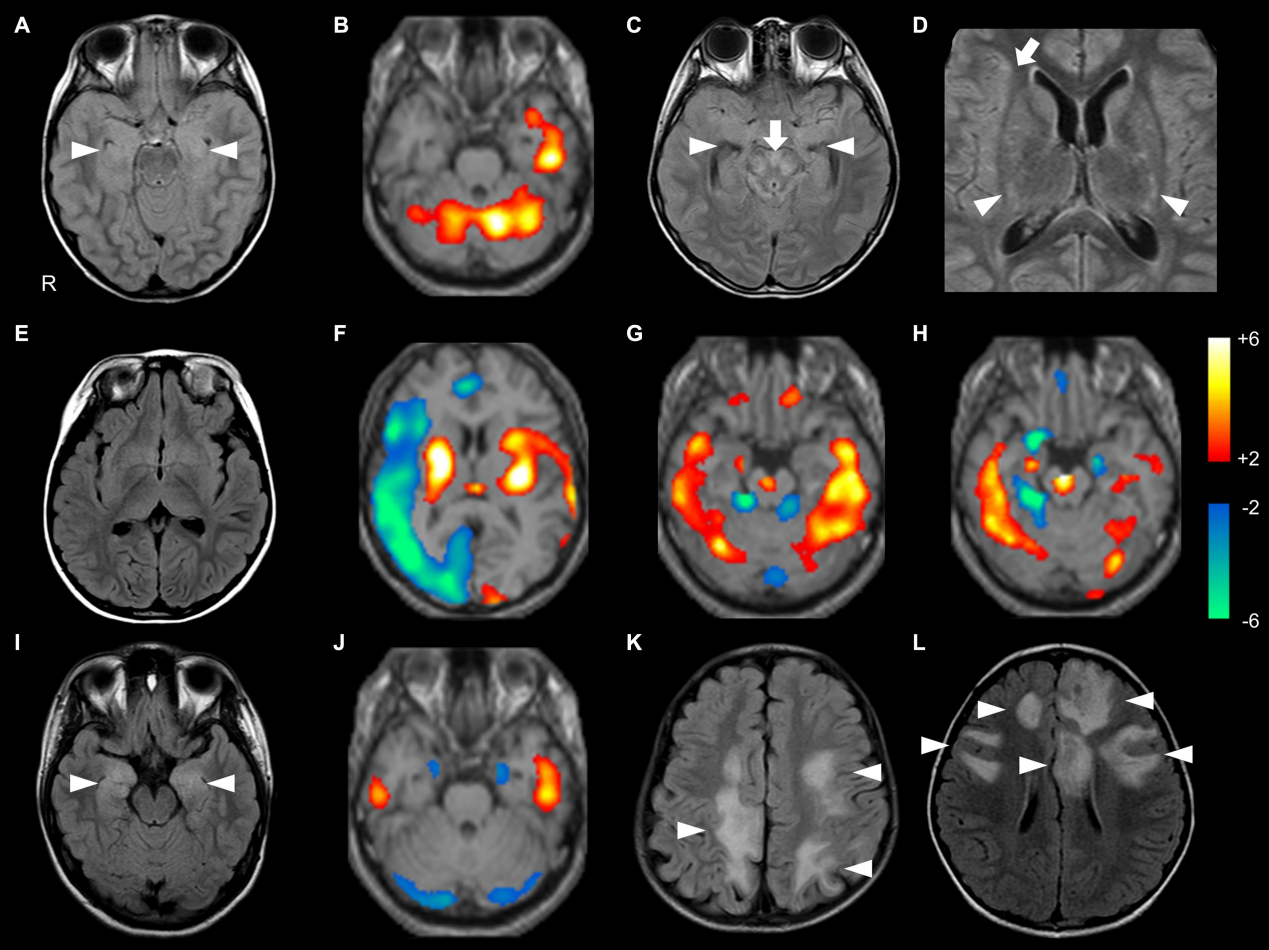
Brain MRI and SPECT findings of probable Ab-negative pediatric autoimmune encephalitis **(A–H)** and major antibody-positive autoimmune encephalitis (NMDARE: **I,J**; MOGAD: **K,L**). In 99mTc-ECD-SPECT images, a regional Z-score of > +2.0 or < −2.0 was considered significant **(B,F–H,J)**. **(A,B)** The same patient. **(A)** FLAIR showed high signal intensities in the bilateral medial temporal lobes (arrowheads). **(B)** ECD-SPECT showed increased blood flow in the left temporal lobe and bilateral cerebellum. **(C,D)** The same patient. FLAIR showed high signal intensities in the bilateral medial temporal (**C**, arrowheads), brainstem (**C**, arrow), basal ganglia (**D**, arrowheads), and right insula (**D**, arrow) with negative anti-glial fibrillary acidic protein antibodies. **(E,F)** The same patient. **(E)** MRI showed no obvious abnormalities. **(F)** ECD-SPECT showed diffusely decreased blood flow in the right frontal, lateral temporal, insular, and occipital regions and increased blood flow in the bilateral basal ganglia, left insular, lateral temporal, and occipital regions. **(G)** Even in a patient without MRI abnormalities, ECD-SPECT showed increased blood flow in the bilateral lateral temporal lobes, cerebellum, frontal lobe, right medial temporal lobe, and brainstem. **(H)** In another patient, ECD-SPECT showed increased blood flow in the bilateral lateral temporal lobes, cerebellum, and brainstem; increased or decreased blood flow in the right medial temporal lobe; and decreased blood flow in the left medial temporal lobe. **(I)** FLAIR demonstrated high signal intensities in the bilateral medial temporal regions in a patient with NMDARE (arrowheads). **(J)** ECD-SPECT exhibited increased blood flow in the bilateral lateral temporal lobes and decreased blood flow in the bilateral medial temporal lobes and cerebellum in an NMDARE patient without MRI abnormalities. **(K,L)** FLAIR showed multiple high signal intensities in the bilateral hemisphere in patients with MOGAD (arrowheads). ECD-SPECT, ethyl cysteinate dimer-single-photon emission computed tomography; FLAIR, fluid-attenuated inversion recovery; MOGAD, myelin oligodendrocyte glycoprotein-associated disease; NMDARE, anti-N-methyl-D-aspartate receptor encephalitis; Probable Ab-negative AE, probable antibody-negative pediatric autoimmune encephalitis.

In the probable Ab-negative AE group, MRI abnormalities were detected in 11 out of 18 (61%) patients with probable Ab-negative AE. Nine (50%) patients had lesions in the medial temporal region (amygdala and hippocampus) (Figures 2A,C). Two patients (11%) had lesions in the frontal lobe, basal ganglia, and insula (Figure 2D).

In the probable Ab-negative AE group, 13 (93%) patients had hyper- or hypo-perfusion on SPECT in the lateral temporal lobe, frontal lobe, and cerebellum. Eleven (79%) patients had hyper- or hypo-perfusion in the medial temporal region (amygdala and hippocampus). Changes in the brainstem were more significant in the probable Ab-negative AE group than in the other two groups (*p* = 0.03). Even in patients with no MRI abnormalities (Figure 2E), all patients in the probable Ab-negative AE group had increased or decreased blood flow in the temporal lobe, frontal lobe, cerebellum, basal ganglia, and others (Figures 2B,F–H). In 4 out of 13 (31%) patients with NMDARE, three showed lesions in the medial temporal lobe (Figure 2I), and one had lesions in the lateral temporal lobe. Two out of three patients with NMDARE exhibited blood flow changes in the bilateral temporal lobes (Figure 2J). All 13 patients with MOGAD presented MRI lesions, with 10 (77%) in the frontal lobe, 7 (54%) in the lateral temporal lobe, and 6 (46%) in the occipital and parietal lobes (Figures 2K,L).

EEG revealed that electroclinical seizures were recorded in 6 out of 17 (35%) patients with probable Ab-negative AE and 3 out of 10 (30%) patients with NMDARE (Table 2).

### 3.3 Treatments

The interval from initial neurological symptoms to first-line immunotherapy was not significantly different among the three groups (Table 1). All patients in the three groups, except for one in the NMDARE group, received IVMP, and more than 75% of patients in each group received IVIG. Following the failure of IVMP and/or IVIG, six patients (one with probable Ab-negative AE, three with NMDARE, and two with MOGAD) underwent PLEX. RTX was administered to 1 out of 18 (6%) patients with probable Ab-negative AE and 4 out of 13 (31%) patients with NMDARE. Oral prednisone was added as maintenance therapy in 14 out of 18 (78%) patients with probable Ab-negative AE, 10 out of 13 (77%) patients with NMDARE, and all patients with MOGAD. Four patients with NMDARE had ovarian teratomas, which were resected immediately after diagnosis.

Regarding intravenous ASMs, all groups received LEV. In addition, MDL and TPL were administered to 7 (39%) and 5 (28%) out of 18 patients with probable Ab-negative AE, respectively. Among 13 patients in the NMDARE group, 4 (31%) and 2 (15%) received MDL and TPL, respectively. The application rates of oral ASMs were significantly higher in patients with probable Ab-negative AE (*p* = 0.01).

### 3.4 Outcomes

**Figure 3:**
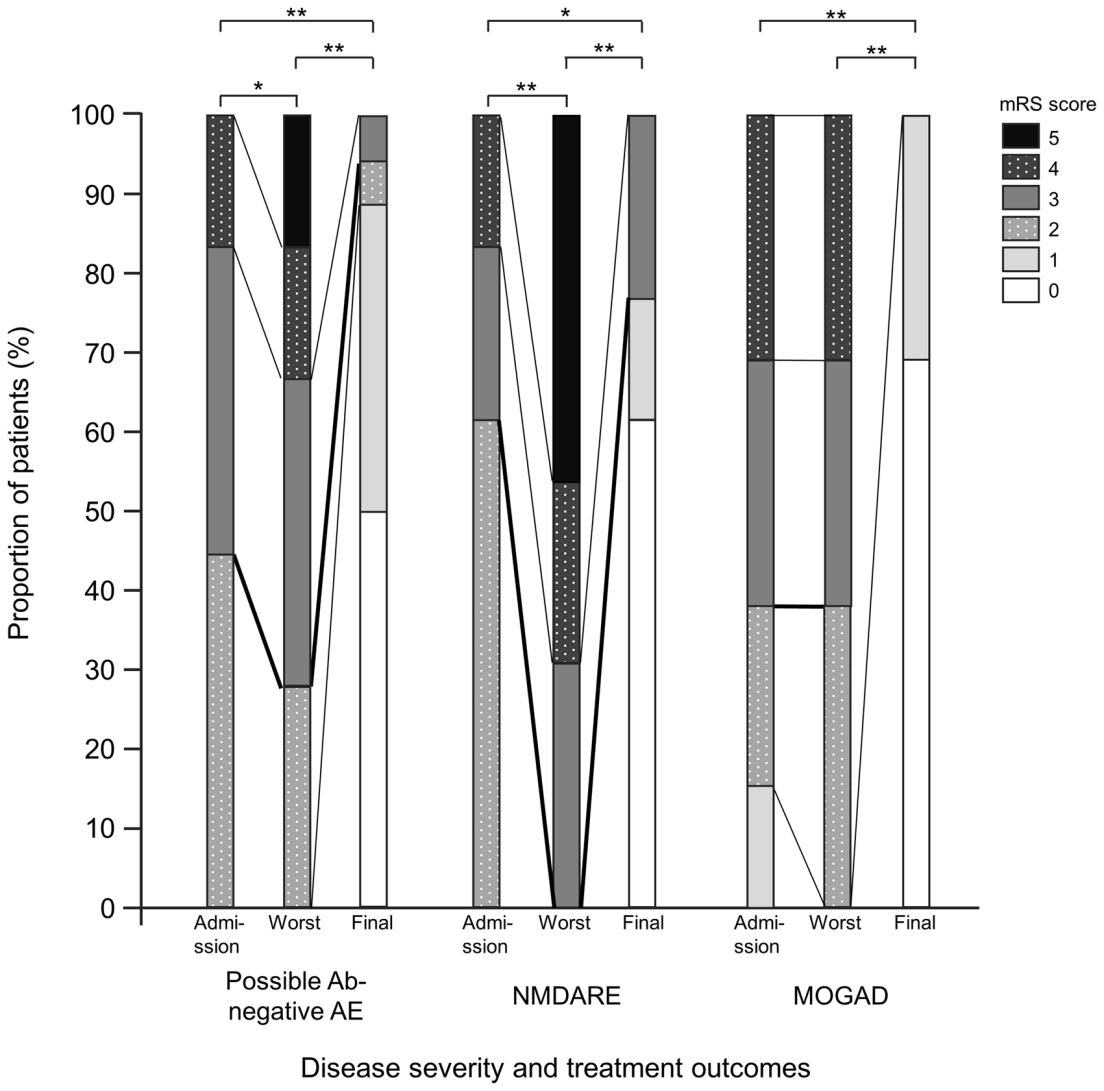
Comparisons between probable Ab-negative pediatric autoimmune encephalitis and major antibody-positive autoimmune encephalitis. Disease severity and treatment outcomes. Black thick lines indicate differences between mRS 2 (good outcome) and 3. Within-group comparisons were performed using the Wilcoxon signed-rank tests. The multiplicity of comparisons among groups was accounted for using the Bonferroni correction. *p < 0.05 and **p < 0.01. MOGAD, myelin oligodendrocyte glycoprotein-associated disease; mRS, modified Rankin Scale; NMDARE, anti-N-methyl-D-aspartate receptor encephalitis; Probable Ab-negative AE, probable antibody-negative pediatric autoimmune encephalitis.

The mRS scores were significantly worse at their worst in the probable Ab-negative AE (*p* = 0.04) and NMDARE (*p* < 0.01) groups than those at admission (Figure 3). Notably, the mRS score significantly improved at the final follow-up compared to admission across all groups (probable Ab-negative AE: *p* < 0.01; NMDARE: *p* = 0.02; and MOGAD: *p* < 0.01). None of the patients with probable Ab-negative AE manifested relapse, whereas 1 out of 13 patients (8%) in the NMDARE group and 7 out of 13 (54%) patients in the MOGAD group relapsed during the follow-up period (*p* < 0.01) (Table 1). The occurrence of autoimmune-associated epilepsy was observed in only one patient (8%) each in the NMDARE and MOGAD groups, but it was observed in seven patients (39%) in the probable Ab-negative AE group; however, this difference was not statistically significant.

## 4 Discussion

Our results suggest the following: (1) Patients in the probable Ab-negative AE group exhibited ASS as initial neurological symptoms significantly more frequently and developed autoimmune-associated epilepsy more frequently than those in the major antibody-positive AE group (not statistically significant, *p* = 0.07), and (2) SPECT within 1 month of disease onset might be a valuable surrogate marker of ongoing neuroinflammation and neuronal dysfunction, even in patients with negative MRI findings.

### 4.1 Acute symptomatic seizures and autoimmune-associated epilepsy with or without autoantibodies in AE

The frequency of ASS in the clinical course of patients with probable Ab-negative AE (78%) was high, consistent with other reports (71–83%) (20, 21, 29). The results for NMDARE (62%) and MOGAD (31%) are also similar to those in other reports [NMDARE: 67–89% (3, 20, 21, 27, 30) and MOGAD: 10–38% (3, 21, 31–33)]. Among the 14 patients in the probable Ab-negative AE group who experienced ASS, 11 had seizures in clusters. Three patients required barbiturate coma therapy for ASS control, highlighting the importance of appropriate identification and control of ASS after admission. Patients with probable Ab-negative AE should receive heightened attention regarding the transition to autoimmune-associated epilepsy (39%) than those with NMDARE or MOGAD (8%). Previous reports have also indicated that the incidence of epilepsy in patients with probable Ab-negative AE was relatively higher (48–61%) (19–21) than in patients with NMDARE (4–14%) (20, 21, 34) or MOGAD (0–14%) (21, 33, 35). Whether early detection and control of ASS may reduce the incidence of autoimmune-related epilepsy needs further investigation, together with the continuation of ASMs after the acute phase and the EEG findings in the course of the disease.

### 4.2 Valuable surrogate marker of SPECT in detecting abnormal alternations in probable Ab-negative AE

SPECT within 1 month of disease onset might be a valuable surrogate marker of ongoing neuroinflammation and neuronal dysfunction, even in patients with negative MRI findings.

Recent reports have also demonstrated that more than half of the patients with AE, regardless of antibodies, show normal brain MRI findings at diagnosis (3, 15, 35). All seven patients with no apparent MRI changes in the probable Ab-negative AE group showed hyper- or hypo-perfusion on SPECT based on eZIS analysis. Therefore, it would be practical to perform SPECT in the acute phase and use indices, such as eZIS analysis, to examine consistency with other modalities (12, 26). In our study, all patients with both NMDARE and MOGAD who performed SPECT also showed evidence of SPECT abnormalities. However, due to the small number of cases, no conclusions can be drawn regarding its usefulness.

Hyper- or hypo-perfusion on SPECT has been reported in a subset of patients with AE, including adult patients (36–38). These findings may indicate active inflammation or neuronal dysfunction of the lesions with changes, even in patients with negative MRI findings (36–38). Psychiatric symptoms may be common with dysfunction in the medial temporal lobes, and altered mental status and cognitive difficulties may be associated with dysfunction of the frontal lobes (1–5, 7). ASSs usually occur in lesions involving the cerebral cortex, and their propagation is considered to involve subcortical structures such as the thalamus, basal ganglia, and brainstem (39–41). The changes in the brainstem were more significant in the probable Ab-negative AE group than in the other two groups in this study. These findings might be related to the higher incidence of ASS in the probable Ab-negative AE. However, multiple lesions were often involved in a single patient, making it difficult to determine the correspondence between symptoms and lesion sites.

Cellucci et al. included brain biopsy as a feature of paraclinical evidence of neuroinflammation; they acknowledged that most pediatric patients with AE do not require brain biopsy (10). Indeed, brain biopsy may be impractical because of its invasiveness in most patients with suspected AE, except in those with suspected Rasmussen encephalitis (25) or malignancy. Therefore, we chose SPECT as an alternative diagnostic criterion for brain biopsies. SPECT within 1 month of disease onset might be a valuable surrogate marker of ongoing neuroinflammation and neuronal dysfunction, even in patients with negative MRI findings. In the recent report on provisional classification criteria (10), Cellucci et al. referred to SPECT as one of the recommended investigations. However, the experience of SPECT in pediatric AE is limited. More prospective research studies into the utilization of SPECT in the workup of antibody-negative AE are needed to conclude the usefulness of SPECT.

### 4.3 Treatments and outcomes

In this study, all patients in the three groups received first-line immunotherapy and may have responded well. Early initiation of first-line immunotherapy in probable Ab-negative AE has shown promising treatment outcomes in several studies (3, 5, 27, 29, 42). Notably, in our study, second-line immunotherapy (RTX) tended to be used less frequently in probable Ab-negative AE (1/18, 6%) than in NMDARE (4/13, 31%). Moreover, the incidence of autoimmune-associated epilepsy in the probable Ab-negative AE group (7/18, 39%) appeared to be higher than that in the major antibody-positive AE group (1/13, 8%). However, these sequelae might be avoided or alleviated by adding second-line immunotherapy at an appropriate time. According to the international consensus recommendations for treating pediatric NMDARE, second-line immunotherapy (preferably RTX over cyclophosphamide) should be considered within 2 weeks of initiating first-line immunotherapy (15). However, in probable Ab-negative AE, where unknown antibodies may be involved or the antibodies themselves may not be implicated, further studies are warranted to determine the appropriate timing and efficacy of second-line immunotherapy.

The mRS scores were significantly worse at their at worst in the probable Ab-negative AE and NMDARE groups than those at admission. In the probable Ab-negative AE and NMDARE group, the mRS of the patients worsened after admission; therefore, we considered the admission explanation to family members to be important.

### 4.4 Limitations

Our study had two limitations. First, the study had a small sample size and a retrospective design. Our hospital is a tertiary care center. Among the 44 patients included in our study, 36 (82%) were transferred from secondary care centers. The main reasons for transfer were poor seizure control, aggravation of psychiatric symptoms, and altered consciousness levels. In this study, all patients received first-line immunotherapy, and we could not evaluate prognostic differences between patients who received immunotherapy and those who did not. In addition, patients with milder symptoms might have been observed without immunotherapy at secondary care centers, leading to a selection bias. Second, this study did not examine other recognized neurological autoantibodies, such as glutamic acid decarboxylase 65, gamma-aminobutyric acid type A receptor, glycine receptor, and dopamine-2 receptor, which may be involved in probable Ab-negative AE. However, we have saved the serum and CSF samples from this study for further analysis in the future.

## 5 Conclusion

Patients with probable Ab-negative AE manifested ASS as their initial neurological symptoms significantly more frequently. They developed autoimmune-associated epilepsy more frequently than those with major antibody-positive AE, which was not statistically significant. SPECT within 1 month of disease onset might be a valuable surrogate marker of ongoing neuroinflammation and neuronal dysfunction, even in patients with negative MRI findings. The newly proposed criteria, with slight modifications, may provide a practical approach for diagnosis accessible to most physicians.

## Data availability statement

The raw data supporting the conclusions of this article will be made available by the authors, without undue reservation.

## Ethics statement

The studies involving humans were approved by the Ethics Committee of Osaka City General Hospital (Approval number: 1508052). The studies were conducted in accordance with the local legislation and institutional requirements. Written informed consent for participation in this study was provided by the participants' legal guardians/next of kin. Written informed consent was obtained from the minor(s)' legal guardian/next of kin for the publication of any potentially identifiable images or data included in this article.

## Author contributions

NaokY: Data curation, Formal analysis, Investigation, Writing – original draft, Writing – review & editing, Conceptualization, Methodology, Validation. TI: Conceptualization, Supervision, Visualization, Writing – original draft, Writing – review & editing, Data curation, Formal analysis, Investigation, Methodology, Validation. IK: Methodology, Writing – review & editing, Conceptualization, Data curation, Formal analysis, Investigation, Validation. NaohY: Data curation, Supervision, Writing – review & editing, Investigation, Validation. MF: Investigation, Methodology, Supervision, Writing – review & editing, Data curation, Validation. MN: Investigation, Supervision, Writing – review & editing, Data curation, Methodology, Validation. HO: Data curation, Formal analysis, Writing – review & editing, Investigation, Methodology, Supervision, Validation. JI: Investigation, Supervision, Writing – review & editing, Data curation, Methodology, Validation. KA: Investigation, Supervision, Validation, Writing – review & editing, Data curation, Methodology. MT: Investigation, Supervision, Writing – review & editing, Data curation, Methodology, Validation. HS: Methodology, Supervision, Validation, Writing – review & editing. SO: Investigation, Methodology, Project administration, Writing – review & editing, Supervision.

## References

[1] Dalmau J Gleichman AJ Hughes EG Rossi JE Peng X Lai M. Anti-NMDA-receptor encephalitis: case series and analysis of the effects of antibodies. Lancet Neurol. (2008) 7:1091–8. doi: 10.1016/S1474-4422(08)70224-2, PMID: 18851928 PMC2607118

[2] Irani SR Bera K Waters P Zuliani L Maxwell S Zandi MS. N-methyl-D-aspartate antibody encephalitis: temporal progression of clinical and Paraclinical observations in a predominantly non-paraneoplastic disorder of both sexes. Brain. (2010) 133:1655–67. doi: 10.1093/brain/awq113, PMID: 20511282 PMC2877907

[3] Hacohen Y Wright S Waters P Agrawal S Carr L Cross H. Paediatric autoimmune encephalopathies: clinical features, laboratory investigations and outcomes in patients with or without antibodies to known central nervous system autoantigens. J Neurol Neurosurg Psychiatry. (2013) 84:748–55. doi: 10.1136/jnnp-2012-303807, PMID: 23175854 PMC3686256

[4] Toledano M Britton JW McKeon A Shin C Lennon VA Quek AM. Utility of an immunotherapy trial in evaluating patients with presumed autoimmune epilepsy. Neurology. (2014) 82:1578–86. doi: 10.1212/wnl.0000000000000383, PMID: 24706013 PMC4013813

[5] Graus F Titulaer MJ Balu R Benseler S Bien CG Cellucci T. A clinical approach to diagnosis of autoimmune encephalitis. Lancet Neurol. (2016) 15:391–404. doi: 10.1016/s1474-4422(15)00401-9, PMID: 26906964 PMC5066574

[6] Dubey D Alqallaf A Hays R Freeman M Chen K Ding K. Neurological autoantibody prevalence in epilepsy of unknown etiology. JAMA Neurol. (2017) 74:397–402. doi: 10.1001/jamaneurol.2016.5429, PMID: 28166327

[7] Dale RC Gorman MP Lim M. Autoimmune encephalitis in children: clinical phenomenology, therapeutics, and emerging challenges. Curr Opin Neurol. (2017) 30:334–44. doi: 10.1097/WCO.0000000000000443, PMID: 28234797

[8] Orozco E Valencia-Sanchez C Britton J Dubey D Flanagan EP Lopez-Chiriboga AS. Autoimmune encephalitis criteria in clinical practice. Neurol Clin Pract. (2023) 13:e200151. doi: 10.1212/CPJ.0000000000200151, PMID: 37124463 PMC10132262

[9] Sakamoto M Matsumoto R Shimotake A Togawa J Takeyama H Kobayashi K. Diagnostic value of an algorithm for autoimmune epilepsy in a retrospective cohort. Front Neurol. (2022) 13:902157. doi: 10.3389/fneur.2022.902157, PMID: 36188368 PMC9518792

[10] Cellucci T Van Mater H Graus F Muscal E Gallentine W Klein-Gitelman MS. Clinical approach to the diagnosis of autoimmune encephalitis in the pediatric patient. Neurol Neuroimmunol Neuroinflamm. (2020) 7:e663. doi: 10.1212/NXI.0000000000000663, PMID: 31953309 PMC7051207

[11] Horita T Inoue T Kuki I Nagase S Yamamoto N Yamada N. A case of bilateral limbic and recurrent unilateral cortical encephalitis with anti-myelin oligodendrocyte glycoprotein antibody positivity. Brain and Development. (2021) 44:254–8. doi: 10.1016/j.braindev.2021.10.011, PMID: 34802814

[12] Yamamoto N Inoue T Kuki I Matsubara K Yamada N Nagase-Oikawa S. A pediatric case of autoimmune glial fibrillary acidic protein Astrocytopathy with unique brain imaging patterns and increased cytokines/chemokines. Brain and Development. (2022) 44:753–8. doi: 10.1016/j.braindev.2022.06.011, PMID: 35840452

[13] Yamada N Kuki I Hattori T Yamamoto N Nagase S Nukui M. Late relapse of anti-N-methyl-D-aspartate receptor encephalitis with Amusia and transiently reduced uptake in 123i-Iomazenil single-photon emission computed tomography. Brain and Development. (2022) 44:558–61. doi: 10.1016/j.braindev.2022.05.003, PMID: 35662527

[14] Nishida H Kohyama K Kumada S Takanashi JI Okumura A Horino A. Evaluation of the diagnostic criteria for anti-Nmda receptor encephalitis in Japanese children. Neurology. (2021) 96:e2070–7. doi: 10.1212/WNL.0000000000011789, PMID: 33653900

[15] Nosadini M Thomas T Eyre M Anlar B Armangue T Benseler SM. International consensus recommendations for the treatment of pediatric NMDAR antibody encephalitis. Neurol Neuroimmunol Neuroinflamm. (2021) 8:e1052. doi: 10.1212/NXI.0000000000001052, PMID: 34301820 PMC8299516

[16] Hacohen Y Banwell B. Treatment approaches for Mog-ab-associated demyelination in children. Curr Treat Options Neurol. (2019) 21:25. doi: 10.1007/s11940-019-0568-z 30671648 PMC6342853

[17] Bruijstens AL Lechner C Flet-Berliac L Deiva K Neuteboom RF Hemingway C. Eu Paediatric Mog consortium consensus: part 1–classification of clinical phenotypes of Paediatric myelin oligodendrocyte glycoprotein antibody-associated disorders. Eur J Paediatr Neurol. (2020) 29:2–13. doi: 10.1016/j.ejpn.2020.10.006, PMID: 33162302

[18] Banwell B Bennett JL Marignier R Kim HJ Brilot F Flanagan EP. Diagnosis of myelin oligodendrocyte glycoprotein antibody-associated disease: international MOGAD panel proposed criteria. Lancet Neurol. (2023) 22:268–82. doi: 10.1016/S1474-4422(22)00431-8, PMID: 36706773

[19] Harvey S Allen NM King MD Lynch B Lynch SA O’Regan M. Response to treatment and outcomes of infantile spasms in down syndrome. Dev Med Child Neurol. (2022) 64:780–8. doi: 10.1111/dmcn.15153 35092693 PMC9303415

[20] Gadian J Eyre M Konstantoulaki E Almoyan A Absoud M Garrood I. Neurological and cognitive outcomes after antibody-negative autoimmune encephalitis in children. Dev Med Child Neurol. (2022) 64:649–53. doi: 10.1111/dmcn.15101 34724211

[21] Woo H Shim Y Chae J-H Kim KJ Lim BC. Seizure evolution and outcome in pediatric autoimmune encephalitis. Pediatr Neurol. (2023) 139:35–42. doi: 10.1016/j.pediatrneurol.2022.11.008, PMID: 36508881

[22] Sakuma H Awaya Y Shiomi M Yamanouchi H Takahashi Y Saito Y. Acute encephalitis with refractory, repetitive partial seizures (AERRPS): a peculiar form of childhood encephalitis. Acta Neurol Scand. (2010) 121:251–6. doi: 10.1111/j.1600-0404.2009.01198.x, PMID: 20028339

[23] Hirsch LJ Gaspard N van Baalen A Nabbout R Demeret S Loddenkemper T. Proposed consensus definitions for new-onset refractory status epilepticus (Norse), febrile infection-related epilepsy syndrome (fires), and related conditions. Epilepsia. (2018) 59:739–44. doi: 10.1111/epi.14016, PMID: 29399791

[24] Horino A Kuki I Inoue T Nukui M Okazaki S Kawawaki H. Intrathecal dexamethasone therapy for febrile infection-related epilepsy syndrome. Ann Clin Transl Neurol. (2021) 8:645–55. doi: 10.1002/acn3.51308, PMID: 33547757 PMC7951105

[25] Kuki I Matsuda K Kubota Y Fukuyama T Takahashi Y Inoue Y. Functional neuroimaging in Rasmussen syndrome. Epilepsy Res. (2018) 140:120–7. doi: 10.1016/j.eplepsyres.2018.01.001 29331846

[26] Mizumura S Kumita S. Stereotactic statistical imaging analysis of the brain using the easy Z-score imaging system for sharing a Normal database. Radiat Med. (2006) 24:545–52. doi: 10.1007/s11604-006-0056-8, PMID: 17058152

[27] Titulaer MJ McCracken L Gabilondo I Armangué T Glaser C Iizuka T. Treatment and prognostic factors for long-term outcome in patients with anti-NMDA receptor encephalitis: an observational cohort study. Lancet Neurol. (2013) 12:157–65. doi: 10.1016/S1474-4422(12)70310-1, PMID: 23290630 PMC3563251

[28] Steriade C Britton J Dale RC Gadoth A Irani SR Linnoila J. Acute symptomatic seizures secondary to autoimmune encephalitis and autoimmune-associated epilepsy: conceptual definitions. Epilepsia. (2020) 61:1341–51. doi: 10.1111/epi.16571, PMID: 32544279

[29] Lee S Kim HD Lee JS Kang H-C Kim SH. Clinical features and treatment outcomes of seronegative pediatric autoimmune encephalitis. J Clin Neurol. (2021) 17:300–6. doi: 10.3988/jcn.2021.17.2.300, PMID: 33835752 PMC8053533

[30] Wang J Lin Z-J Liu L Xu H-Q Shi Y-W Yi Y-H. Epilepsy-associated genes. Seizure. (2017) 44:11–20. doi: 10.1016/j.seizure.2016.11.030 28007376

[31] Shen C-H Zheng Y Cai M-T Yang F Fang W Zhang Y-X. Seizure occurrence in myelin oligodendrocyte glycoprotein antibody-associated disease: a systematic review and Meta-analysis. Mult Scler Relat Disord. (2020) 42:102057. doi: 10.1016/j.msard.2020.102057, PMID: 32222694

[32] Liu K Sun S Cui J Zhang L Zhang K Zhang L. Seizures in myelin oligodendrocyte glycoprotein antibody-associated disorders and related immune factors. Seizure. (2021) 92:216–20. doi: 10.1016/j.seizure.2021.09.011, PMID: 34600301

[33] Montalvo M Khattak JF Redenbaugh V Britton J Sanchez CV Datta A. Acute symptomatic seizures secondary to myelin oligodendrocyte glycoprotein antibody-associated disease. Epilepsia. (2022) 63:3180–91. doi: 10.1111/epi.17424, PMID: 36168809 PMC10641900

[34] Liu X Guo K Lin J Gong X Li A Zhou D. Long-term seizure outcomes in patients with autoimmune encephalitis: a prospective observational registry study update. Epilepsia. (2022) 63:1812–21. doi: 10.1111/epi.17245, PMID: 35357695

[35] Suleiman J Dale RC. The recognition and treatment of autoimmune epilepsy in children. Dev Med Child Neurol. (2015) 57:431–40. doi: 10.1111/dmcn.12647, PMID: 25483277

[36] Kimura N Kumamoto T Takahashi Y. Brain perfusion Spect in limbic encephalitis associated with autoantibody against the glutamate receptor epsilon 2. Clin Neurol Neurosurg. (2014) 118:44–8. doi: 10.1016/j.clineuro.2013.12.006, PMID: 24529228

[37] Ohta K Seki M Dalmau J Shinohara Y. Perfusion IMP-SPECT shows reversible abnormalities in GABA(B) receptor antibody associated encephalitis with Normal MRI. Brain Behav. (2011) 1:70–2. doi: 10.1002/brb3.14, PMID: 22399086 PMC3236545

[38] Heine J Prüss H Bartsch T Ploner C Paul F Finke C. Imaging of autoimmune encephalitis–relevance for clinical practice and hippocampal function. Neuroscience. (2015) 309:68–83. doi: 10.1016/j.neuroscience.2015.05.037 26012492

[39] McCormick DA Contreras D. On the cellular and network bases of epileptic seizures. Annu Rev Physiol. (2001) 63:815–46. doi: 10.1146/annurev.physiol.63.1.815, PMID: 11181977

[40] Norden AD Blumenfeld H. The role of subcortical structures in human epilepsy. Epilepsy Behav. (2002) 3:219–31. doi: 10.1016/s1525-5050(02)00029-x 12662601

[41] Vercueil L Hirsch E. Seizures and the basal ganglia: a review of the clinical data. Epileptic Disord. (2002) 4 Suppl 3:S47–54. doi: 10.1684/j.1950-6945.2002.tb00545.x, PMID: 12495874

[42] Sabanathan S Abdel-Mannan O Mankad K Siddiqui A Das K Carr L. Clinical features, investigations, and outcomes of pediatric limbic encephalitis: a multicenter study. Ann Clin Trans Neurol. (2022) 9:67–78. doi: 10.1002/acn3.51494, PMID: 35015932 PMC8791799

